# MDMA, methamphetamine, and CYP2D6 pharmacogenetics: what is clinically relevant?

**DOI:** 10.3389/fgene.2012.00235

**Published:** 2012-11-12

**Authors:** Rafael de la Torre, Samanta Yubero-Lahoz, Ricardo Pardo-Lozano, Magí Farré

**Affiliations:** ^1^Human Pharmacology and Clinical Neurosciences Research Group, Neurosciences Research Program, IMIM-Hospital del Mar Medical Research InstituteBarcelona, Spain; ^2^Department of Experimental and Health Sciences, Universitat Pompeu FabraBarcelona, Spain; ^3^Department of Pharmacology, Therapeutics and Toxicology, Universitat Autònoma de BarcelonaBarcelona, Spain

**Keywords:** MDMA, CYP2D6, methamphetamine, pharmacogenetics, ecstasy

## Abstract

*In vitro* human studies show that the metabolism of most amphetamine-like psychostimulants is regulated by the polymorphic cytochrome P450 isozyme CYP2D6. Two compounds, methamphetamine and 3,4-methylenedioxymethamphetamine (MDMA), were selected as archetypes to discuss the translation and clinical significance of *in vitro* to *in vivo* findings. Both compounds were chosen based on their differential interaction with CYP2D6 and their high abuse prevalence in society. Methamphetamine behaves as both a weak substrate and competitive inhibitor of CYP2D6, while MDMA acts as a high affinity substrate and potent mechanism-based inhibitor (MBI) of the enzyme. The MBI behavior of MDMA on CYP2D6 implies that subjects, irrespective of their genotype/phenotype, are phenocopied to the poor metabolizer (PM) phenotype. The fraction of metabolic clearance regulated by CYP2D6 for both drugs is substantially lower than expected from *in vitro* studies. Other isoenzymes of cytochrome P450 and a relevant contribution of renal excretion play a part in their clearance. These facts tune down the potential contribution of CYP2D6 polymorphism in the clinical outcomes of both substances. Globally, the clinical relevance of CYP2D6 polymorphism is lower than that predicted by *in vitro* studies.

## Introduction

Amphetamine-type stimulants (ATS) make up a group of substances comprised of synthetic stimulants including amphetamine, methamphetamine, methcathinone, and ecstasy-group substances [e.g., 3,4-methylenedioxymethamphetamine (MDMA) and its analogues]. According to the latest report published by UNODC, ATS such as “ecstasy” and methamphetamine now rank as the world's second most widely abused drug type after cannabis (UNODC, 2011).

*In vitro* human studies show that the metabolism of most psychostimulants belonging to this class of compounds is regulated by the polymorphic cytochrome P450 isozyme CYP2D6. In addition, some of them behave both as substrates and inhibitors of CYPD6 and several other CYP isozymes (Wu et al., 1997; see Table 1). The gene (CYP2D6) environment (drug use, gender, ethnicity…) interaction has been evaluated in drug users in order to evaluate: (1) intensity of drug effects, (2) susceptibility to acute toxicity episodes and fatalities, (3) susceptibility to drug dependence, (4) contribution to drug induced neurotoxicity, and (5) drug-drug pharmacological interactions.

The fact that this polymorphic enzyme partially regulates metabolic disposition leads us to postulate that acute toxicity, drug abuse and dependence as well as, in some cases, long-term neurotoxicity could be influenced by CYP2D6 genetics (Sellers and Tyndale, 2000; de la Torre and Farré, 2004; Perfetti et al., 2009). Specifically, it was postulated that:

1. Subjects carrying genotypes which lead to enzymatic functional phenotypes should display an increased risk of drug abuse proportionate to their genotype (homozygous vs. heterozygous) and absolute level of enzyme activity.
2. Subjects carrying genotypes which lead to enzymatic functional phenotypes should display an increased risk of neurotoxicity proportionate to their genotype and absolute level of enzyme activity if the underlying mechanism is unrelated to a metabolic bioactivation.
3. Subjects carrying genotypes which lead to non-functional enzyme should experience greater risk of toxicity to a drug which is not metabolically inactivated, and might be less likely to acquire drug-taking behavior.

The present review will examine available clinical data to determine to what extent these postulates have been confirmed. Due to the fact that ATS are a broad class of compounds encompassing a number of substances, and that scant *in vivo* data from human studies are available for most of them (Wu et al., 1997), the review will focus mainly on the following two: MDMA (ecstasy) and methamphetamine. They will serve as archetypes in order to discuss the translation and clinical significance of *in vitro* to *in vivo* findings. Both compounds were chosen based on their differential interaction with CYP2D6 and their abuse prevalence in society.

## CYP2D6, MDMA, and methamphetamine: background considerations

CYP2D6 accounts for only a small percentage of total hepatic cytochrome P450 (1–2%), yet it is responsible for the metabolism of approximately 20–30% of marketed pharmaceuticals, including tricyclic antidepressants, selective serotonin reuptake inhibitor antidepressants, opioids, and antipsychotic, antiemetic, antiarrhythmic, and amphetamine-like drugs (Ingelman-Sundberg, 2005). *CYP2D6* exhibits a marked genetic polymorphism—over 70 alleles (www.cypalleles.ki.se/cyp2d6.htm) have been described whose combination leads to four phenotypes: poor, intermediate, extensive, and ultrarapid metabolizers (PM, IM, EM, and UM, respectively). Subjects with a PM phenotype lack two functional alleles; those with an IM have one reduced-activity allele and one non-functional allele or two reduced-activity alleles; whereas EM individuals have one or two functional alleles; and the UM phenotype is associated with gene duplications of functional alleles, with an increased protein expression (Zanger et al., 2004; Bogni et al., 2005). About 5–10% of Caucasians are PM, presenting a metabolic deficiency in CYP2D6 activity (Sachse et al., 1997).

**Figure 1:**
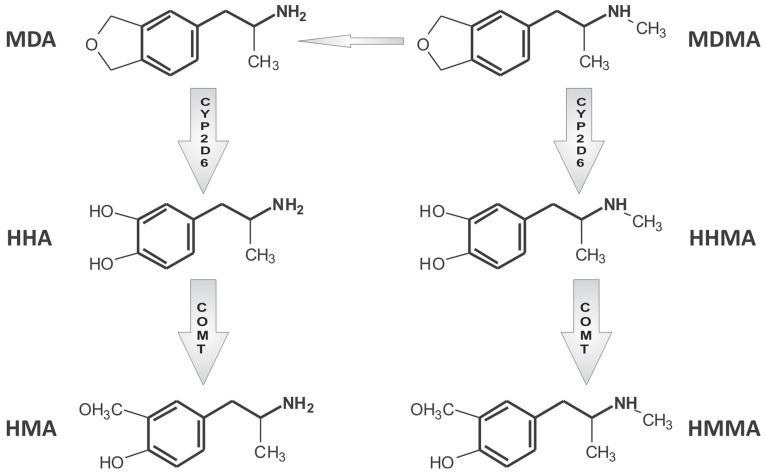
**Simplified scheme of MDMA main metabolic pathways.** For a more detailed description, readers are referred to de la Torre et al. (2004). MDMA (3,4-methylendioxymethampetamine), MDA (3,4-methylendioxyampetamine), HHMA (3,4-dihydroxy methamphetamine), HMMA (3-methoxy-4-hydroxymethamphetamine), HHA (3,4-dihydroxyamphetamine), and HMA (3-methoxy-4-hydroxyamphetamine).

**Figure 2:**
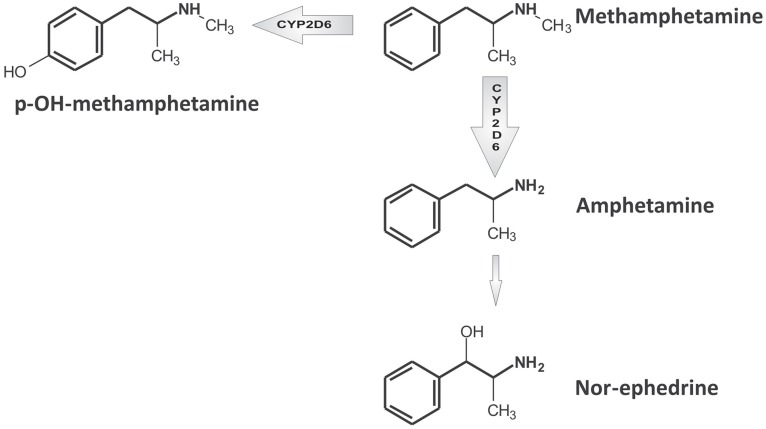
**Simplified scheme of methamphetamine main metabolic pathways.** For a more detailed description, readers are referred to Shima et al. (2006).

CYP2D6 regulates MDMA O-demethylenation leading to the formation of 3,4-dihydroxymethamphetamine (HHMA) and the 4-hydroxylation of methamphetamine (pholedrine) (see Figures 1 and 2). Both compounds are, therefore, substrates of the same enzyme although the rates by which they are oxidized differ markedly. MDMA oxidation takes place at almost 100 times the rate of methamphetamine oxidation (see Table 1) (Lin et al., 1997). While methamphetamine is both a substrate and competitive inhibitor of CYP2D6, MDMA acts as a substrate and potent mechanism-based inhibitor (MBI) of the enzyme (Delaforge et al., 1999).

**Table 1:** **Interaction of amphetamine-like and related psychostimulants with CYP2D6: affinity and inhibitory capacity**.

**Amphetamine derivatives**	**Ki (μM)^*a*^**	**Km (μM)**
±3,4-Methylenedioxymethamphetamine (MDMA)	0.6 ± 0.6	2.2 ± 1.6^*b*^
±3,4-Methylenedioxyamphetamine (MDA)	1.8 ± 1.0	11.6 ± 5.4^*b*^
±-3,4-methylenedioxyethylamphetamine (MDE)		2.6 ± 1.4^*b*^
+Amphetamine	26.5 ± 1.5	
+Methamphetamine	25.0	39.6 ± 4.7^*c*^
±-2-Methoxyamphetamine	11.5 ± 0.5	
±-3-Methoxyamphetamine	17.5 ± 2.5	
±-4-Methoxyamphetamine (PMA)	24 ± 6	29.3 ± 4.4^*d*^
±-4-Hydroxyamphetamine	195 ± 45	
±-4-Hydroxymethamphetamine	60 ± 10	
*p*-Methoxymethamphetamine (PMMA)		4.6 ± 1.0^*e*^
4-methoxy-Nethylamphetamine (M-NEA)		19.5 ± 1.4^*d*^
N-butylamphetamine (NBA)		3.7 ± 0.3^*d*^
4-methoxy-N-butylamphetamine (M-NBA)		11.9 ± 1.8^*d*^
±-2,4,6-Trimethoxyamphetamine (2,4,6-TMA)	33 ± 12	
±-3,4,5-Trimethoxyampthetamine (3,4,5-TMA)	128 ± 3	
benzodioxolyl-butanamine (BDB)		0.8 ± 0.1^*f*^
N-methyl-benzodioxolyl-butanamine (MBDB)		1.0 ± 0.02^*g*^
4′-Methyl-α -pyrrolidinopropiophenone (MPPP)		9.8 ± 2.5^*h*^
4′-methoxy-α -pyrrolidinopropiophenone (MOPPP)		9.9 ± 2.5^*i*^
3′,4′-methylenedioxy-α -pyrrolidinopropiophenone (MDPPP)		13.5 ± 1.5^*j*^

a (Wu et al., 1997).

b (Kreth et al., 2000).

c (Lin et al., 1997).

d (Bach et al., 1999).

e (Staack et al., 2004). Calculated as pmol/min/pmol P450.

f (Meyer et al., 2009b). Calculated as pooled human liver microsomes (pHLM 20 mg microsomal protein/mL, 400 pmol total P450/mg protein).

g (Meyer et al., 2009a). Calculated as pooled human liver microsomes (pHLM 20 mg microsomal protein/mL, 400 pmol total P450/mg protein).

h (Springer et al., 2003a). Calculated as pmol/min/pmol P450.

i (Springer et al., 2003b). Calculated as pmol/min/pmol P450.

j (Springer et al., 2003c). Calculated as pmol/min/pmol P450.

The east and south–east regions of Asia home to about one-third of the global population, have one of the most strongly established markets for ATS in the world, primarily for methamphetamine (UNODC, 2011). This is of relevance since the CYP2D6*10 allelic variant, which encodes a hypofunctional enzyme, is carried by approximately 75% of the Asian population. An *in vitro* study comparing catalytically the CYP2D6*10 allelic variant vs. CYP2D6*1 (wild type), which is more prevalent in Caucasians, showed that the ratios of intrinsic clearance (Vmax/Km) of *1 to *10 for MDMA O-demethylenation was 123, and for methamphetamine 67, for the p-hydroxylation 157 of the N-demethylation (Ramamoorthy et al., 2001). This almost 100-fold difference in intrinsic clearance for both drugs, depending on the allelic variant considered, is of significance in the interpretation of clinical data. Findings observed in Caucasian populations with respect to the impact of *CYP2D6* polymorphism on drug effects are not always reproduced in Asian ones. This lack of reproducibility is due to the distribution of extreme phenotypes (UM and PM) in these populations. Globally, however, the rate of metabolic disposition of CYP2D6 substrates is slower in Asians.

Another aspect to be considered is the common belief that amongst drug abuser populations genotypes are distributed according to the same patterns as the general population. In the case of CYP2D6, assuming that PM subjects have a higher tendency to accumulate drug in the body, either these subjects discontinue drug consumption or autoregulate it by taking lower doses. Due to the relatively small size of the populations evaluated in clinical studies (in terms of guaranteeing a fair representation of all genotypes), the number of subjects carrying extreme genotypes is usually quite small. In fact, the authors of the present review believe that PM subjects are underrepresented in MDMA and other amphetamine-like compound user populations probably because of the acute effects experienced. This bias has to be taken into consideration when interpreting clinical data.

## MDMA pharmacology and metabolic disposition

MDMA is a psychostimulant drug that displays effects related to amphetamine-type drugs plus a number of distinctive ones (closeness to others, facilitation to interpersonal relationship, and empathy) that have been named by some authors as entactogen properties. MDMA is a potent releaser and/or reuptake inhibitor of presynaptic serotonin (5-HT), dopamine (DA), and norepinephrine (NE). These actions result from the interaction of MDMA with the membrane transporters involved in neurotransmitter reuptake and vesicular storage systems. MDMA is a mild inhibitor of monoamine oxidase (MAO) and also has direct action on several types of receptors including the 5-HT2 receptor, the M1 muscarinic receptor, the α2-adrenergic receptor, and the histamine H1 receptor.

The most frequent effects after MDMA administration are euphoria, well-being, happiness, stimulation, increased energy, extroversion, feeling close to others, increased empathy, increased sociability, enhanced mood, and mild perceptual disturbances. In addition, cardiovascular related somatic symptoms, autonomic effects (dry mouth, sweating, tremor, mydriasis tremor, jaw clenching, and restlessness), and moderate derealization have been observed (de la Torre et al., 2004).

MDMA induced acute toxic effects are related to its pharmacologic actions. Hyponatremia is an uncommon complication associated with inappropriate antidiuretic hormone (SIADH) secretion and excessive water intake. Fulminant hepatitis and hepatic necrosis have been described (Henry et al., 1992; de la Torre et al., 2004). Chronic use of MDMA is linked to a progressive neurodegeneration of the serotonergic neurotransmission system (Green et al., 2003).

Two main pathways are involved in MDMA metabolic clearance: (1) O-demethylenation partially regulated by CYP2D6 followed by catechol-O-methyltransferase (COMT)-catalyzed methylation (HMMA) and/or glucuronide/sulfate conjugation; and (2) N-dealkylation leading to 3,4-methylenedioxyamphetanmine (MDA), further subject to similar metabolic reactions as MDMA (O-demethylenation and O-methylation) (see Figure 1). MDMA metabolic clearance accounts for about 75% of plasma clearance and 30% of its metabolism is regulated by CYP2D6 (de la Torre et al., 2000; Segura et al., 2005).

## MDMA CYP2D6 mechanism-based inhibition and the phenocopying phenomenon

As previously stated, whilst MDMA is metabolized by CYP2D6 it is also a potent MBI of the enzyme (de la Torre et al., 2000; Farré et al., 2004; Heydari et al., 2004; Yang et al., 2006). MBI occurs shortly after a single recreational dose, inactivating most hepatic CYP2D6 within 2 h, and returning to a basal level of CYP2D6 activity after at least 10 days (O'Mathúna et al., 2008; Yubero-Lahoz et al., 2011). This phenomenon is associated with a decrease in the amount of effective enzyme so that recovery of activity depends on its *de novo* synthesis (Liston et al., 2002). In MBI there is a rapid phenocopying to apparent PM status after a single dose of MDMA, which signifies that within 2 h subjects display the PM phenotype after drug intake, irrespective of their original genotype. Previous clinical trials have reported that the phenocopying phenomenon was observed in 67% of male subjects (O'Mathúna et al., 2008) and in 100% of the females (Yubero-Lahoz et al., 2011). Therefore, recreational MDMA users (including those who take repeated doses in the same session) are exposed to a higher probability of relative overdose and an increased risk of suffering adverse effects from CYP2D6 substrates (Farré et al., 2004).

## MDMA and CYP2D6 pharmacogenetics

Most research evaluating the potential impact of CYP2D6 pharmacogenetics in MDMA pharmacology has been focused on acute effects. Preliminary *in vitro* studies showing that MDMA was a CYP2D6 substrate raised the possibility that subject carriers of allelic variants leading to the PM phenotype for CYP2D6 might be at increased risk of acute toxicity episodes and higher abuse liability (Tucker et al., 1994; Henry and Hill, 1998; de la Torre et al., 1999). Conversely, since long-term neurotoxicity is believed to be mediated by metabolites formed after methylenedioxyphenyl ring-opening by CYP2D6 (de la Torre and Farré, 2004; Jones et al., 2005), PM might be protected against chronic toxicity (Perfetti et al., 2009). However, toxicological data do not seem to fully support these expectations since in a series of acute intoxications, high plasma MDMA concentrations have been reported although an overrepresentation of genotypes (homozygous for the *3 and *4 allelic variants examined) leading to the PM phenotype was not found (O'Donohoe et al., 1998; Schwab et al., 1999; Gilhooly and Daly, 2002). Unfortunately, few data are available concerning the clinical pharmacology of MDMA in PM individuals. A previous study reported that PM (homozygous *4/*4) subjects display increased plasma concentrations, and an increased risk of hyperthermia, after a single dose of MDMA. A similar observation has been reported for another methylenedioxyamphetamine derivative,3,4-methylenedioxyethylamphetamine (MDE) (Kreth et al., 2000). Concerning MDMA, due to CYP2D6 autoinhibition and, therefore, the phenomenon of phenocopying towards the PM phenotype, effects experienced by EM subjects (*1/*1, *n* = 6, and *1/*4 *n* = 3) after two consecutive doses of the drug (Farré et al., 2004; de la Torre et al., 2005) are similar to those of PM ones (*4/*4). Other studies have reported that drug side effects are related individuals with low-activity of CYP2D6 (EM/IM category, comprising the following genotypes: *2/*9, *1/*10, *1/*41, *2/*41, *2/*35, *35/*35, and *35/*41) displaying an increased induction of plasma hypo-osmolality, hyponatremia, and increased plasma antidiuretic hormone (vasopressin) after MDMA consumption (Aitchison et al., 2012). Moreover, the PM/IM (*4/*29, *5/*41, and *6/*41) or the IM/IM (*41/*41) genotypes were related to a greater degree of increase in plasma cortisol concentration than the other CYP2D6 after MDMA intake (Wolff et al., 2012).

The formation of tioether adducts with quinones resulting from the auto-oxidation of the MDMA catechol type metabolites HHMA and HHA (3,4-dihydroxyamphetamine) is one of the hypotheses for MDMA induced neurotoxicity. These compounds can easily enter into redox cycling, generating radical oxygen species, which are the underlying mechanism of MDMA neurotoxicity. Genetic polymorphisms in CYP2D6 and catechol-O-methyltransferase, the combination of which are major determinants of steady-state levels of HHMA and HMMA, probably explain the interindividual variability seen in the recovery of N-acetyl-cysteinyl adducts from urine (N-Ac-5-Cys-HHMA and N-Ac-5-Cys-HHA). The recovery was marginally related to the CYP2D6 genotype among EM subjects (one vs. two functional alleles) (*p* < 0.1) and to the COMT *val158met* genotype (*p* < 0.1) of subjects. The recovery of N-Ac-5-Cys-HHMA was 2-fold higher among *met/met* subjects compared with the value for the *val/val* subjects (Perfetti et al., 2009).

## MDMA drug−drug interactions

MDMA, once taken, is not selective to CYP2D6 and interacts with several isozymes of P450. In fact, the contribution of CYP2D6 to MDMA metabolism has been reported to be less than 30% (Segura et al., 2005). Thus, several other P450 isoenzymes such as CYP1A2 and, to a lesser extent, CYP2B6 and CYP3A4 have the capacity to contribute to the microsomal oxidative metabolism of MDMA and MDA A recent study showed that while CYP2D6 was inhibited by MDMA, CYP1A2 increased its activity (Yubero-Lahoz et al., 2012). Another clinical trial showed there was a conversion from MDMA to HHMA *in vivo*, despite the CYP2D6 inhibition by paroxetine, suggesting alternative metabolic pathways (Segura et al., 2005). Other enzymes may, therefore, become more predominant once CYP2D6 is inhibited which could further contribute to MDMA metabolic disposition.

The administration of inhibitors of CYP2D6 activity can influence the metabolism of MDMA, and in turn MDMA can inhibit drugs metabolized by CYP2D6. Previous administration of antidepressants with CYP2D6 inhibitory actions, such as paroxetine, reboxetine, or duloxetine, produce 15–30% of MDMA concentrations, but decrease concentrations of its metabolite HMMA by 40–50% (Segura et al., 2005; Farré et al., 2007; Hysek et al., 2011, 2012). Moreover, the pharmacological effects of MDMA are decreased, probably due to competition for the uptake transporter decreasing MDMA entry in neurons.

CYP2D6 is also the source of a number of drug–amphetamine interactions because it regulates the biotransformation of many therapeutic drugs. Antiretroviral drugs (ritonavir, a known CYP2D6 inhibitor) and MAO inhibitors have been reported to be the main cause of life threatening interactions with MDMA (Henry and Hill, 1998; de la Torre et al., 1999; Papaseit et al., 2012).

## Methamphetamine pharmacology and metabolic disposition

Methamphetamine is an indirect sympathomimetic agent, similar in structure to amphetamine. Nevertheless, an added N-methyl group confers increased lipid solubility, allowing for more rapid diffusion into the central nervous system. Methamphetamine effects derive from their interaction with a number of neurotransmitter systems; primarily with the dopaminergic but also with serotonergic, noradrenergic, and glutamatergic systems. Acute adverse effects including cardiovascular and psychoactive ones are related to an excess of neurotransmitters. As previously explained, long-term methamphetamine induced effects are the result of a neurodegeneration of the dopaminergic system (Schep et al., 2010).

There are three main biotransformation pathways (see Figure 2) involved in methamphetamine metabolic clearance: (1) N-demethylation to produce amphetamine, (2) aromatic hydroxylation producing 4-hydroxymethamphetamine (pholedrine) with both reactions partially regulated by CYP2D6 (Lin et al., 1997), and (3) beta-hydroxylation to produce norephedrine. Metabolic clearance represents more than 50% of total plasma clearance (Cook et al., 1993).

## Methamphetamine and CYP2D6 pharmacogenetics

A review of the current literature for genetic-association studies of methamphetamine use disorders, including 38 studies and 39 genes, showed that 18 genes were found to have a significant genotypic, allelic, and/or haplotypic association. Among these genes was CYP2D6 which was associated with methamphetamine dependence (Bousman et al., 2009). Of particular relevance was a report in which a total of 202 patients with methamphetamine dependence and 337 controls in a Japanese population were genotyped for CYP2D6*1, *4, *5, *10, and *14. A significant association of the CYP2D6 genotype with methamphetamine dependence (*p* = 0.03) was reported. There were fewer patients carrying the hypofunctional alleles *10 and *14 alleles than in the control population, and in this population there were no PMs. IMs of CYP2D6 were significantly fewer among methamphetamine-dependent subjects than in controls (*p* = 0.02), with an odds ratio of 0.62 (95% confidence interval: 0.51–0.76). A potential conclusion of this study is that a lower CYP2D6 activity seems to confer some degree of protection against methamphetamine dependence (Otani et al., 2008).

In a study performed in Caucasians it was observed that EM (*n* = 8) and PM (*n* = 3) subjects administered with methamphetamine by the oral route (10 mg) displayed a similar area under the curve for plasma methamphetamine concentrations, despite the fact that p-hydroxymethamphetamine was only observed in PM subjects. PM subjects appeared to be more sensitive to the slope of plasma methamphetamine concentrations in several measurements of subjective effects. The data suggest that brain methamphetamine concentrations (in the absence of differences in plasma concentrations) are higher in PM subjects or that they have a steeper concentration-response relationship (Sellers and Tyndale, 2000).

Methamphetamine use may induce the following physical effects: anorexia, hyperactivity, dilated pupils, flushing, restlessness, dry mouth, bruxism, headache, cardiovascular alterations in heart rate, and blood pressure. It may cause rhabdomyolysis which has been associated with mortality. In a series of 18 autopsies genetic susceptibility to rhabdomyolysis was examined. Mutations of the following genes were studied: ryanodine receptor 1 (RYR 1), carnitine palmitoyltransferase II (CPT II), very long-chain acyl-CoA dehydrogenase (VLCAD), and CYP2D6. The conclusion was that there was no obvious relationship between the genetic mutations observed in this study and rhabdomyolysis (Matsusue et al., 2011).

Neuropsychological alterations seen in many methamphetamine users are often unrelated to its lifetime consumption or length of abstinence. In a series of 52 methamphetamine users the contribution of CYP2D6 polymorphism to variability observed in long-term effects was studied. EM subjects showed worse overall neuropsychological performance and were three times as likely to be cognitively impaired as IMs/PMs. Apparently, a more efficient metabolic disposition of methamphetamine is associated with a poorer cognitive performance. It has also been suggested that metabolism may generate metabolic species involved in the underlying mechanism of neurotoxicity (Cherner et al., 2010).

## Methamphetamine drug—drug interaction

The administration of inhibitors of CYP2D6 activity can influence the metabolism of methamphetamine, and methamphetamine can inhibit the metabolism of CYP2D6 substrates. The number of published drug-interaction studies with this class of substances is very scarce. Previous administration of bupropion, a known CYP2D6 inhibitor, produces a large increase of methamphetamine concentrations, and a reduction in amphetamine ones. The pharmacological effects of methamphetamine (cardiovascular and euphoria-like ones) were decreased by bupropion (Newton et al., 2005, 2006).

Antiretroviral drugs (ritonavir, a known CYP2D6 inhibitor) have been reported to be the main cause of life-threatening interactions with methamphetamine (Hales et al., 2000).

## Concluding remarks

The involvement of CYP2D6 polymorphism in the metabolic clearance of both MDMA and methamphetamine leads to the speculation that it should have an impact on acute and long-term drug toxicity and drug taking behavior.

Concerning acute effects, those subject carriers of alleles with a reduced functionality are at higher risk, for both MDMA and methamphetamine, of experiencing heightened pharmacological effects. Moreover, in combination with some environmental factors this may lead to acute toxicity episodes including death. Irrespective of the initial dose, the following one results in the MBI of MDMA and phenocopying to the PM phenotype thus diluting the variability incorporated by the genetic polymorphism and, consequently, putting all subjects at risk of acute effects. With respect to methamphetamine, because a large portion of drug users are of Asian origin, and carriers of the lower functionality allele *10, most of these subjects should also experience increased effects.

Concerning the relevance of CYP2D6 polymorphism on drug abuse, preliminary data from methamphetamine suggest that an increased CYP2D6 functionality may lead to an increased abuse of the substance. An observation that is more relevant for methamphetamine, with a higher abuse liability, than MDMA.

Regarding neurotoxicity, only in the case of MDMA may a metabolic bioactivation be involved in long-term neurotoxic effects. Theoretically those subject carriers of CYP2D6 functional alleles (including those carriers of duplications) and with a low COMT activity should be the most efficient in generating metabolic neurotoxic species and, consequently, the most vulnerable to neurotoxicity. Again the MBI of MDMA should be taken into consideration after repeated doses.

Metabolic clearance of both methamphetamine and MDMA ranges from 50% to 75%, there is, therefore, a relevant contribution of renal excretion in plasma clearance. The fraction of metabolic clearance regulated by CYP2D6 is lower than 50% for both drugs and other isozymes of cytochrome P450 contribute to their clearance. Both factors combine with catalytic activities, and MBI behavior in case of MDMA, to tune down the potential contribution of CYP2D6 polymorphism in clinical outcomes of both substances. Although MDMA and methamphetamine are the most consumed ATS many substances of this group are substrates of CYP2D6. Thus, in possible future reports on other substances it would not be surprising to find that the CYP2D6 polymorphism has a strong role in the clinical outcome of drug users. The difficulty in performing controlled clinical studies with drug users stratified as a function of drug metabolizing polymorphisms, limits the evaluation of their clinical impact.

In summary, the genetic polymorphism of CYP2D6 and co-administration of CYP2D6 inhibitors may have less impact on the risk of acute toxicity than previously thought, whereas the role of metabolism by other cytochrome P450 enzymes and renal excretion assumes greater importance with regard to systemic exposure to unchanged drug. Globally, the clinical relevance of CYP2D6 polymorphism is lower than that predicted by *in vitro* studies.

### Conflict of interest statement

The authors declare that the research was conducted in the absence of any commercial or financial relationships that could be construed as a potential conflict of interest.
